# The relationship between clinics and the venom of the causative Amazon pit viper (*Bothrops atrox*)

**DOI:** 10.1371/journal.pntd.0008299

**Published:** 2020-06-08

**Authors:** Ana Maria Moura-da-Silva, Jorge Carlos Contreras-Bernal, Sarah Natalie Cirilo Gimenes, Luciana Aparecida Freitas-de-Sousa, José Antonio Portes-Junior, Pedro da Silva Peixoto, Leo Kei Iwai, Valéria Mourão de Moura, Pedro Ferreira Bisneto, Marcus Lacerda, Iran Mendonça da Silva, Luiz Carlos de Lima Ferreira, Sâmella Silva de Oliveira, Fan Hui Wen, Jacqueline de Almeida Gonçalves Sachett, Wuelton M. Monteiro

**Affiliations:** 1 Instituto Butantan, São Paulo, Brazil; 2 Diretoria de Ensino e Pesquisa, Fundação de Medicina Tropical Dr. Heitor Vieira Dourado, Manaus, Brazil; 3 Escola Superior de Ciências da Saúde, Universidade do Estado do Amazonas, Manaus, Brazil; 4 Instituto de Matemática e Estatística, Universidade São Paulo, São Paulo, Brazil; 5 Programa de Pós-graduação em Recursos Naturais da Amazônia, Universidade Federal do Oeste do Pará, Santarém, Pará, Brazil; 6 Programa de Pós-Graduação em Zoologia, Universidade Federal do Amazonas, Manaus, Brazil; 7 Instituto Leônidas & Maria Deane, Manaus, Brazil; 8 Diretoria de Ensino e Pesquisa, Fundação Alfredo da Matta, Manaus, Brazil; Institut de Recherche pour le Développement, BENIN

## Abstract

Snake venoms are complex mixtures of proteins with toxic activities, with many distinct isoforms, affecting different physiological targets, comprised in a few protein families. It is currently accepted that this diversity in venom composition is an adaptive advantage for venom efficacy on a wide range of prey. However, on the other side, variability on isoforms expression has implications in the clinics of human victims of snakebites and in the efficacy of antivenoms. *B*. *atrox* snakes are responsible for most of the human accidents in Brazilian Amazon and the type and abundance of protein families on their venoms present individual variability. Thus, in this study we attempted to correlate the individual venom proteome of the snake brought to the hospital by the patient seeking for medical assistance with the clinical signs observed in the same patient. Individual variability was confirmed in venoms of the 14 snakes selected for the study. The abundance of each protein family was quite similar among the venom samples, while the isoforms composition was highly variable. Considering the protein families, the SVMP group presented the best correlation with bleeding disorders and edema. Considering individual isoforms, some isoforms of venom metalloproteinase (SVMP), C-type lectin-like toxins (CTL) and snake venom serine proteinases (SVSP) presented expression levels that with statistically significant positive correlation to signs and symptoms presented by the patients as bleeding disorders, edema, ecchymosis and blister formation. However, some unexpected data were also observed as the correlation between a CTL, CRISP or LAAO isoforms with blister formation, still to be confirmed with a larger number of samples. Although this is still a small number of patient samples, we were able to indicate that venom composition modulates clinical manifestations of snakebites, to confirm at the bedside the prominent role of SVMPs and to include new possible toxin candidates for the development of toxin inhibitors or to improve antivenom selectiveness, important actions for the next generation treatments of snakebites.

## Author summary

*Bothrops atrox* is a snake of major medical importance in the Amazon. Its venom is specialized to kill preys in the nature, especially because of coagulotoxic and proteolytic activities. *B*. *atrox* envenomings cause local inflammation and, in a significant proportion, systemic manifestations, namely bleeding disorders. These signs and symptoms are caused by the various toxins present in the venom of this snake, which act in the organism by different mechanisms. It is not known to what extent the composition of the venom that was inoculated by the snake that caused the envenoming can influence the patient’s clinical condition. To study this subject, this work correlated the constituents of the venom with the clinical manifestations of hospitalized patients, taking advantage of the fact that many patients bring the snake responsible for the bite. The abundance of each toxin family was similar among the venom samples, but the variants composition of each toxin was highly variable. Considering the protein families, a group named metalloproteases (SVMP) presented the best correlation with bleeding disorders and edema. Some variants of venom SVMPs, and other toxin families, such as C-type lectin-like toxins (CTL) and snake venom serine proteinases (SVSP) presented correlation to signs and symptoms presented by the patients as bleeding disorders, edema, ecchymosis and blister formation. Our results show that venom composition modulates clinical manifestations of snakebites.

## Introduction

Snakebite is a neglected tropical disease with high incidence in Brazil, especially in the Amazon region [1]. *Bothrops atrox* is the snake species responsible for approximately 90% of the snakebites in Brazilian Amazon [2]. Unclottable blood, a predictor of systemic bleeding, is the commonest hemostatic disorder in the envenomation, while local signs ranges from pain and swelling at the site of bite minutes after the event, to intense signs and symptoms at the bitten limb, with blistering and tissue necrosis. Secondary infection, compartmental syndrome, and extensive necrosis can lead to temporary or permanent disability of the bitten limb. Spontaneous systemic bleeding and acute renal failure are common complications from *B*. *atrox* envenomings [3,4]. However, the occurrence of each sign/symptom is variable among the patients. In a recent study, 54% of patients of Manaus, in the Brazilian Amazon, presented unclottable blood at admission [5], while systemic bleeding are reported in around 15% of the cases [6]. Several factors have been associated with the envenomations’ characteristics and severity, such as the patient’s condition, pre-hospital treatments and the time before antivenom therapy [7]. Aspects related to the snake involved in the envenomation, such as their ontogenetic stage, have also been correlated to patients’ signs and symptoms, possibly caused by the individual variability in snake venom composition [8].

In *B*. *atrox* snakes collected at Brazilian Amazon, venoms are predominately composed by snake venom metalloproteinase (SVMP) followed by C-type lectin-like toxins (CTL), snake venom serine proteinases (SVSP), phospholipases A_2_ (PLA_2_), cysteine-rich secretory proteins (CRISP), L-amino acid oxidases (LAAO) and other minor components [9–11]. It is widely accepted that the spectrum of the snakebite envenomation depends on the additive or synergistic action of these toxins. Venom-induced coagulopathy has been correlated to thrombin-like SVSPs and procoagulant SVMPs that activates coagulation factors II and X [10,12]. In addition, CTLs or acidic PLA_2_s have an anticoagulant effect by inhibiting components of the coagulation cascade [13]. SVMPs classes PI and P-III such as Atroxlysin-I [14,15] and Batroxrhagin [16] cause damage in vascular endothelium resulting in local and/or systemic bleeding and contributing to the ischemia on tissues adjacent to the bite. SVMPs and PLA_2_s display direct proinflammatory activity [17,18] or induce the release of endogenous proinflammatory activators of TLR pathways [19]. Cytotoxic toxins acting directly on different cell types are also present as myotoxic PLA_2_s [20] or proapoptotic LAAOs [21] and SVMPs [22,23], enhancing the local damage induced by the hemostatic disturbances and proinflammatory effects of venom toxins. However, these functional assumptions must be taken with some concerns. Several structurally-related isoforms are included within each protein family, but in spite of structural similarity, they may display different biological activities and target distinct physiological pathways [24]. The functional variability of snake venom components is a great adaptive advantage for snakes enabling the capture of a wider prey variety but has important consequences for human envenomings.

The abundance of each toxin family and their isoforms varies in venoms of different specimens of *B*. *atrox* snakes [25], according to snake ontogeny [26,27], geographical distribution [9,28] and habitats occupied by a single population [11]. Clinically, intraspecific differences could impact on clinical outcomes and in the neutralizing capacity of the antivenoms [11,12,28]. In this study, we attempted to correlate the venom composition and the abundance of each component of venom in samples collected from *B*. *atrox* snakes brought to the hospital by the patients, to the clinical manifestations presented by the patient on the healthcare unit.

## Material and methods

### Patients

**Fig 1:**
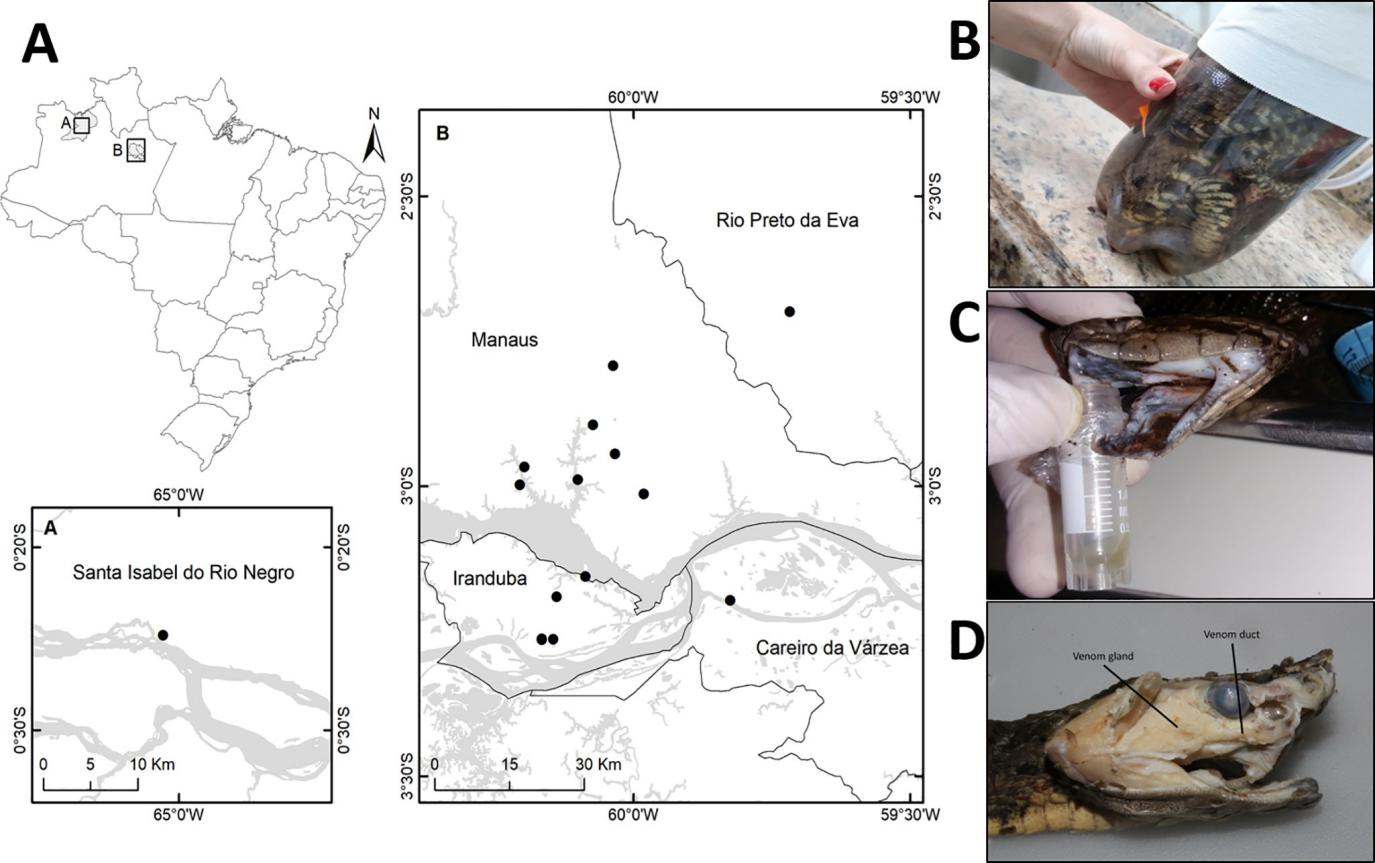
Area of the snakebites and venom sample extraction from *Bothrops atrox* specimens. Patients were bitten nearby Manaus city, State of Amazonas, Brazil. (A). Usually they bring the snakes in bottles (B). Venom was extracted from the snake fangs (C) or in some specimens, venom gland was exposed and venom samples collected by puncturing the gland lumen (D). Map produced using QGIS, Open Source Geospatial Foundation Project http://qgis.osgeo.org.

We included snakebites occurring at Manaus, Brazilian Amazon (Fig 1A), attended at *Fundação de Medicina Tropical Dr*. *Heitor Vieira Dourado* (FMT-HVD), from January to December 2017. Eligible patients presented clinical signs of *Bothrops* envenomation and brought the snake responsible for the envenomation, which was identified as *B*. *atrox*. On admission, epidemiological and clinical information was collected using a standardized questionnaire. Envenomation was classified as mild, moderate, or severe, according to the Brazilian Ministry of Health guidelines [29]. Edema was classified as absent, mild (affecting 1±2 segments), moderate (affecting 3±4 segments) and severe (affecting more than 5 limb segments). Presence of pain, local bleeding, ecchymosis, necrosis and systemic bleeding were also recorded. Compartment syndrome was diagnosed by an experienced physician by serial physical examinations and intramuscular pressure measurement. Coagulopathy was defined as an unclottable blood from the Lee-White clotting time method [6]. Patients were treated according the Brazilian Ministry of Health protocols. All patients were evaluated for at least 48 hours.

### Venom extraction and chromatographic characterization

Venom was extracted individually from the snakes brought by the patients (Fig 1B) and only living animals or snakes killed less than 8 hours before hospital admission were included. Venom was collected from the fangs (Fig 1C) by massages in the venom gland region. In some specimens, the venom gland was exposed and venom samples collected by puncturing the gland lumen (Fig 1D). After extraction, venom samples were conserved freeze-dried and dissolved before use in 50 mM Tris buffer, pH 7.2. Protein concentration was estimated using Bradford reagent and BSA dilutions as a standard curve. Individual venom samples (2 mg) were fractionated by reversed-phase high-performance liquid chromatography (RP-HPLC) following previously described methods [30]. The chromatographic profiles obtained were analyzed based on a previously performed standard chromatogram, in which components present in every fraction were characterized by mass spectrometry [11].

### Proteomic characterization

Each venom sample (50 μg) was reduced and alkylated before treatment with trypsin solution (0.2 μg/μL), as previously described [31]. The tryptic digests were desalted using Empore C18-SD 4mm/1mL column (Supelco, UK). Peptide samples were resuspended in 0.1% FA (formic Acid) and each sample was analyzed in duplicate using an EASY-nLC system (Thermo Scientific) coupled to LTQ-Orbitrap Velos mass spectrometer (Thermo Scientific). The peptides were loaded onto a C18 PicoFrit column (C18 PepMap, 75 μm id × 10 cm, 3.5 μm particle size, 100 Å pore size; New Objective, Ringoes, NJ, USA) and separated with a gradient from 100% mobile phase A (0.1% FA) to 34% phase B (0.1% FA, 95% ACN) during 60 min, 34%–95% in 15 min and 5 min at 95% phase B at a constant flow rate of 250 nL/min. The LTQ-Orbitrap Velos was operated in positive ion mode with data-dependent acquisition. The full scan was obtained in the Orbitrap with an automatic gain control (AGC) target value of 10e6 ions and a maximum fill time of 500 ms. Each precursor ion scan was acquired at a resolution of 60,000 FWHM in the 400–1500 m/z mass range. Peptide ions were fragmented by CID MS/MS using a normalized collision energy of 35. The 20 most abundant peptides were selected for MS/MS and dynamically excluded for 30s. All raw data were assessed in the Xcalibur software (Thermo Scientific). Analysis have been carried out at BIOMASS facility (CEFAP-USP, São Paulo, Brazil). Tandem mass spectra were processed and searched against an in house database composed by the full-length precursor proteins predicted from the transcriptomes of five specimens of *B*. *atrox* [25], using the search tools Mascot (Matrix Science, London, UK; version 2.6.2) and X! Tandem [(The GPM, thegpm.org; version X! Tandem Alanine (2017.2.1.4)]. The Scaffold package (Scaffold_4.9.0, Proteome Software Inc., Portland, OR) was used to validate MS/MS-based peptide and protein identifications. Protein identification was based on the presence of at least two proteotypic peptides relating to each venom protein isoform. Quantitative values were expressed for protein families as normalized total spectral counts of all isoforms included in the same group and for isoforms, as normalized exclusive unique spectrum counts corresponding to peptides of a given protein entry present in the database.

### Data analyses

Multiple cross-correlation analyses between variables were performed. Due to the categorical nature of the “signs/symptoms” variable, and the small sample size, Spearman Rank Correlation Tests were employed in all cross-correlation calculations. Also, due to the small sample size, formal statistical testing is not to be interpreted as rigorous quantitative confidence of the results discussed, but rather a piece of supporting information corroborating the biological interpretation. Accordingly, we, therefore, relax the usual 95% confidence interval used in biostatistics and adopt 90% confidence interval in all tests.

### Ethical clearance

Ethical approval for human information collection was obtained from the *Fundação de Medicina Tropical Doutor Heitor Vieira Dourado* (approval number 1302174/2016.). Written informed consent was obtained from the patient or their guardians for minors. Snake manipulation was approved by the FMT-HVD Animal Ethical Committee (001552/2017.011) and registered in SISGEN under process A3A5599.

## Results and discussion

### Patients’ signs and symptoms

During the period of the study, 32 patients brought the snake involved in the envenomation for identification at the FMT-HVD hospital. Sixteen patients were not included because they were brought more than 8 hours after the bite. Venom was successfully extracted from the other 16 specimens in quantities that allowed compositional characterization. Two patients presenting “dry bite” were further excluded. The remaining 14 patients were included in the study, recorded their major signs and symptoms of the envenomation, and the characterization of the venom composition of the perpetrating snake was made. Most of the patients were bitten on the foot (10), and took from 40 min to 6:30 h to receive health care. Three applied tourniquets at the bitten limb. All patients presented edema (14), followed by pain (13) and local bleeding (6). One patient developed blistering, and 4 evolved to secondary infection after 48 hours of follow-up (3 with abscess and 1 with cellulitis) and 2 had necrosis. Blood was unclottable in 8 cases, and one patient manifested systemic bleeding. Clinical severity was considered moderate in 9 patients and mild in 4; only one patient presented a severe envenomation. There were no deaths (Table 1). It is important to note that the estimation of the amount of venom injected to the patients, by quantification either in circulation or in the tissues, would be of high value for this study. However, these tests are not currently used at the hospital and serum samples before antivenom administration were available from only four patients, thus the obtained values not included in the study.

**Table 1:** Epidemiological and clinical characteristics of the cases.

Snake code	BATX3	BATX5	BATX8	BATX9	BATX10	BATX13	BATX15	BATX18	BATX24	BATX25	BATX27	BATX28	BATX30	BATX32
**Gender**	M	M	M	M	M	M	M	M	M	M	M	M	M	M
**Age (years)**	40	14	89	55	33	55	42	53	33	14	45	31	6	23
**Municipality**	Rio Preto da Eva	Iranduba	Manaus	Manacapuru	Manaus	Iranduba	Manaus	Manaus	Santa Isabel do Rio Negro	Iranduba	Manaus	Manaus	Careiro da Várzea	Manaus
**Anatomical site**	Foot	Hand	Foot/hand	Foot	Leg	Foot	Foot	Foot	Foot	Foot	Hand	Foot	Leg	Foot
**Time elapsed bite/assistance**	1h30min	1h30min	2hs	40min	2hs	1h	2hs30min	4h30min	4hs	2hs30min	6hs30min	2hs15min	5hs	3hs
**Previous history of snakebite**	Yes	No	No	No	No	No	Yes	No	No	No	No	No	No	No
**Preadmission procedures**	No	No	No	No	No	No	Tourniquet	No	No	No	Tourniquet	Tourniquet	No	No
Severity of envenomation*	2	2	2	1	2	2	2	1	2	1	2	2	3	1
**Unclottable blood**^§^	1	0	1	1	0	1	1	1	0	0	0	1	0	1
Edema*	2	2	3	2	2	2	2	2	1	1	2	2	3	1
**Local bleeding**^§^	1	1	1	0	0	0	1	0	1	0	1	0	0	0
**Local ecchymosis**^§^	0	0	1	0	0	0	1	0	0	0	0	1	0	0
**Pain**^§^	0	1	1	1	1	1	1	1	1	1	1	1	1	1
**Systemic hemorrhage**^§^	0	0	0	1	0	0	0	0	0	0	0	0	0	0
**Blistering**^§^	0	0	0	0	0	0	1	0	0	0	0	0	0	0
**Complications after 48 hs**	0	0	compartment syndrome	abscess	0	cellulitis	necrosis and abscess	0	necrosis and abscess	0	0	0	0	0

*For severity of envenomation and edema: 1 = mild, 2 = moderate, 3 = severe;

^§^For unclottable blood, local bleeding, ecchymosis, pain, systemic hemorrhage, blistering and complications after 48 hs: 0 = absent, 1 = present.

### Characterization of venom composition

**Fig 2:**
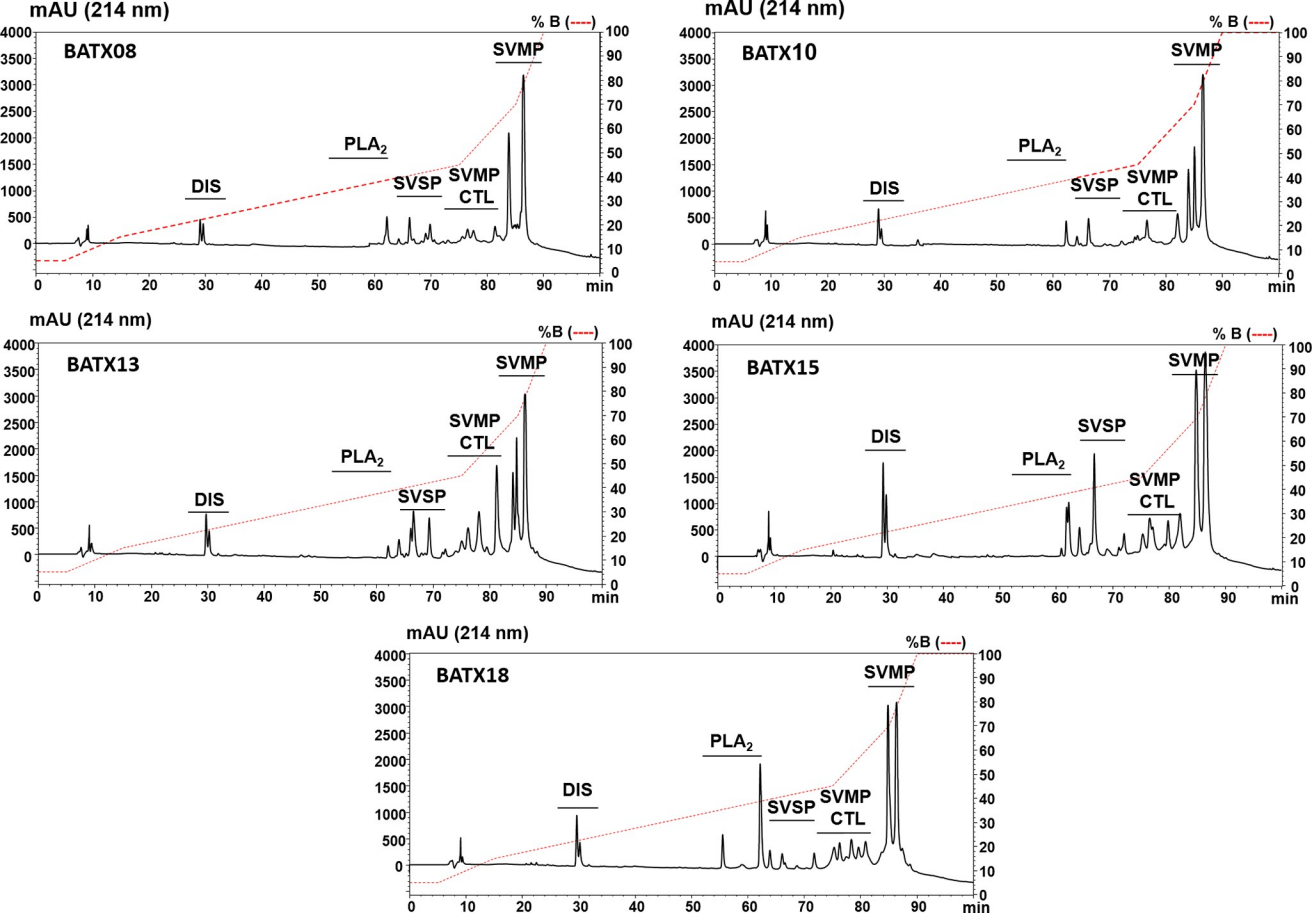
Comparison of the chromatographic profiles of venom samples from the snakes. Samples containing 2 mg of crude venom were fractionated by RP-HPLC as described in Methods section. Regions eluting disintegrins (Dis), phospholipases A_2_ (PLA_2_), serine proteinases (SVSP), C-type lectin-like (CTL) and metalloproteinases (SVMP) are annotated and were identified as characterized in a previous study [11].

Variability in venom composition was confirmed in five specimens with sufficient venom to perform RP-HPLC chromatography. As shown in Fig 2, each sample displayed different chromatographic profile, evidencing the individual variability in the venom composition of snakes involved in each envenomation. The common characteristics of all venoms were the elution of the highest peaks after 85 minutes, which is characteristic to the elution of SVMPs and consistent with the predominance of this toxin family in the venoms of *B*. *atrox* snakes collected in different areas of Brazilian Amazon [32]. Nevertheless, the shape and abundance of each peak in the region indicate that different SVMP isoforms are dominant in the venom of each snake. Moreover, variability in the expression of other protein families was indicated as higher peaks were observed in the regions that elute CTLs, SVSPs and PLA_2_s in venoms of BATX 13, BATX 15 and BATX 18 snakes, respectively (Fig 2).

**Fig 3:**
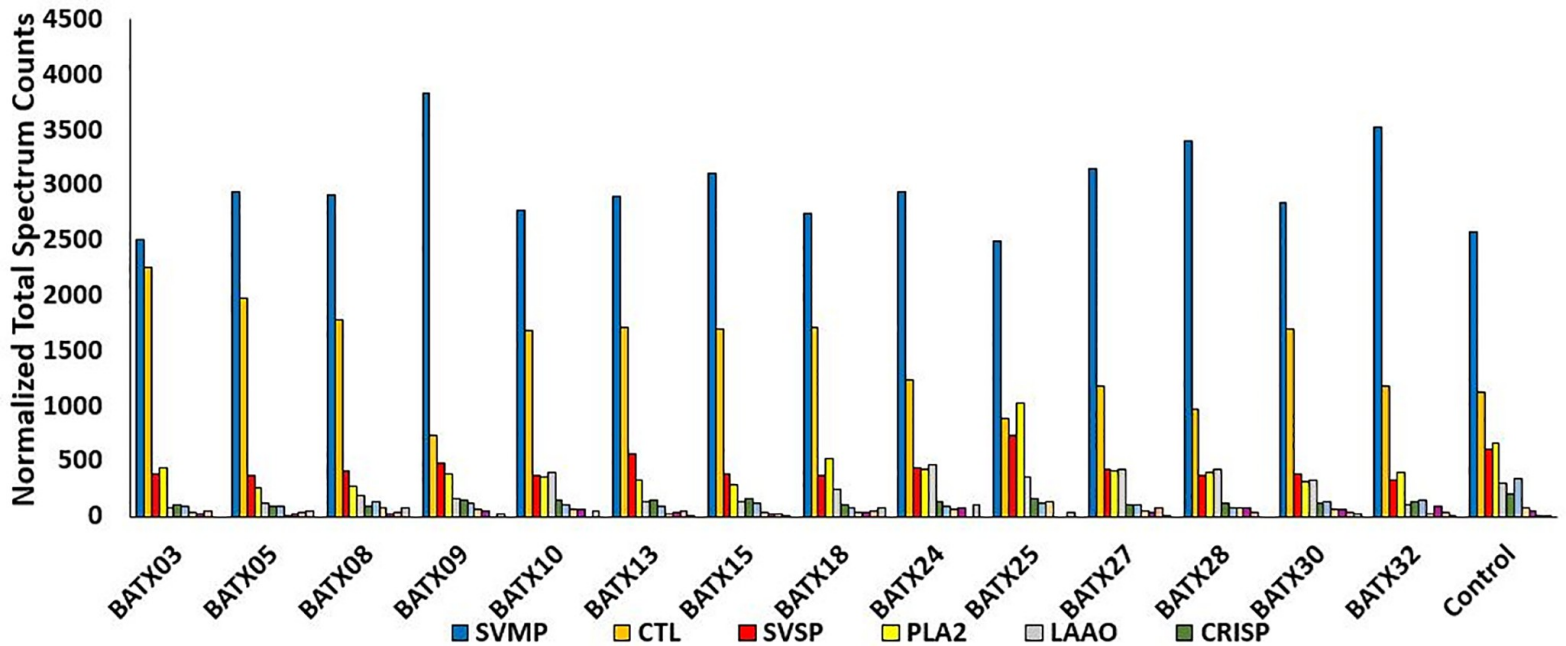
Proteomic profile of the individual venom samples. Relative expression indicated by the values of normalized total spectrum counts of toxins identified in the venoms of snakes brought to the hospital by 14 patients. Control is the venom of a live *B*. *atrox* specimen maintained under captivity at FMT-HVD serpentarium. Toxin isoforms were grouped according to the toxin families: SVMP—snake venom metalloproteinase; CTL—C-type lectin; SVSP—snake venom serine proteinase; PLA_2_—phospholipase A_2_; LAAO—L-amino acid oxidase; CRISP—cysteine-rich secretory protein; PDE—phosphodiesterase; NUC—nucleotidase; VEGF—vascular endothelial growth factor; HYAL—hyaluronidase; NGF—nerve growth factor.

Next, we evaluated by shotgun proteomics the venom composition and variability of expression levels of each protein family among the venom samples (detailed proteomics data is shown in S1 and S2 Tables). In these analyses, a pool of venoms from live *B*. *atrox* snakes from the same region, maintained under captivity, was used as control (Fig 3). All venoms shared the presence of 11 protein families: SVMP, CTL, SVSP, LAAO, PLA_2_, CRISP, phosphodiesterases (PDE), nucleotidases (NUC), venom vascular endothelial growth factors (VEGF), nerve growth factors (NGF), and hyaluronidases (HYAL). In all samples, there was a predominance of SVMPs and CTLs, followed by SVSPs, PLA_2_s and LAAO, with smaller amounts of CRISPs, NUCs, PDEs, VEGFs, NGFs, and HYALs, similar to previous results [9,30]. However, the expression levels of protein groups differed among the venoms. For example, BATX 9 venom showed the highest levels of SVMPs and the lowest of CTLs (Fig 3).

Venom variability was also assessed by the label-free quantification of the isoforms in each pool of venom was based on the *exclusive unique spectrum counts* to avoid the redundancy due to the sequence similarity of isoforms present in each protein group. This approach was possible and reliable since we used as databank a comprehensive masterset containing 150 complete sequences obtained by transcriptomics of venom glands from five *B*. *atrox* specimens [25]. The numbers of exclusive unique spectra counted for each isoform are shown in S1 Table. In Table 2 we highlight the great variability in the expression levels of isoforms among the venoms. BATXSVMPI5 and BATXSVMPIII28 were the most abundant isoforms in all venom samples, even though, with differences in their expression levels. These sequences are from two hemorrhagic toxins recently isolated from *B*. *atrox* venoms named Atroxlysin-Ia [14] and Batroxrhagin [16], respectively, that degrade extracellular matrix and display proinflammatory activity (Almeida et al., submitted). Other toxins as BATXSVMPIII1, BATXCRISP1, and BATXPDE1 are also present above the average in all venom samples. Most of the isoforms presented great variability in their expression levels among the venoms. Good examples are BATXPLA3, BATXCLT28, BATXSVMPIII16 and BATXLAAO2 expression levels among the venoms. Also interesting is the BATX 32 venom that presents above average levels of most SVMPs and lower levels of isoforms from other protein families (Table 2). This picture is in agreement with our previous data showing that Atroxlysin-Ia and Batroxrhagin are core function toxins highly preserved and widely expressed in *B*. *atrox* individual venoms while other isoforms are more likely to variability attempting to a functional reservoir for snake adaptivity [25]. However, the low and uniform expression of SVSP isoforms in these venom samples was not expected.

**Table 2:** Normalized Exclusive Unique Spectrum Count of predominant isoforms in venoms*.

Isoforms	BATX 3	BATX 5	BATX 8	BATX 9	BATX 10	BATX 13	BATX 15	BATX 18	BATX 24	BATX 25	BATX 27	BATX 28	BATX 30	BATX 32
**CTL**														
**BATXCTL9**	20.43	19.04	18.72	11.90	15.61	15.57	17.57	14.46	13.93	17.41	12.57	10.28	15.23	10.62
**BATXCTL23**	0.00	0.00	0.00	0.00	0.00	0.00	13.66	4.57	0.00	0.00	0.00	0.00	0.00	0.00
**BATXCTL28**	30.10	22.84	20.80	8.50	21.47	19.47	26.35	4.57	19.73	12.06	10.90	10.28	18.08	1.93
**BATXCTL39**	25.80	14.28	17.68	7.65	18.54	19.47	9.76	12.94	18.57	12.06	18.44	16.82	13.32	7.72
**PLA**_**2**_														
**BATXPLA2**	32.25	2.86	2.08	0.85	13.66	1.95	3.90	22.84	5.80	38.84	0.00	24.30	4.76	0.97
**BATXPLA3**	0.00	19.99	16.64	9.35	0.00	13.63	20.49	0.00	20.89	0.00	0.00	0.00	17.13	0.00
**BATXPLA5**	26.88	20.94	23.92	22.95	22.45	6.81	21.47	28.16	34.82	17.41	18.44	24.30	3.81	30.90
**BATXPLA6**	23.65	0.00	0.00	11.90	19.52	16.55	0.00	15.22	0.00	21.43	20.11	12.15	19.04	10.62
**SVMP**														
**BATXSVMPI5**	53.76	45.68	46.79	35.69	60.51	35.04	53.68	35.78	48.74	49.56	36.04	46.73	41.88	18.35
**BATXSVMPIII1**	35.48	41.88	31.20	33.99	40.99	33.09	40.01	26.64	32.50	36.17	27.66	34.58	24.75	42.49
**BATXSVMPIII2**	13.98	13.32	3.12	12.75	8.78	12.65	10.74	25.88	11.61	12.06	25.14	23.37	2.86	32.83
**BATXSVMPIII5**	19.35	15.23	16.64	20.40	20.49	15.57	19.52	15.99	20.89	14.73	15.09	15.89	13.32	20.28
**BATXSVMPIII9**	11.83	24.75	20.80	35.69	4.88	24.33	25.37	35.78	18.57	18.75	28.50	29.91	22.84	29.93
**BATXSVMPIII16**	7.53	8.57	0.00	25.49	4.88	25.31	9.76	26.64	18.57	4.02	31.01	25.24	14.28	47.31
**BATXSVMPIII18**	23.65	20.94	23.92	17.00	31.23	19.47	0.00	21.31	18.57	12.06	27.66	9.35	19.99	4.83
**BATXSVMPIII24**	31.18	32.36	45.75	11.05	22.45	4.87	44.89	25.88	3.48	20.09	5.87	34.58	39.97	32.83
**BATXSVMPIII27**	0.00	2.86	0.00	20.40	5.86	27.25	0.00	24.36	9.28	8.04	24.31	10.28	0.95	33.80
**BATXSVMPIII28**	79.56	74.24	73.83	62.04	82.95	56.45	69.29	49.48	81.24	66.97	61.18	69.17	63.77	49.25
**SVSP**														
**BATXSVSP10**	16.13	19.04	15.60	4.25	12.69	15.57	14.64	1.52	13.93	10.72	10.90	12.15	14.28	1.93
**BATXSVSP20**	11.83	14.28	15.60	16.15	10.74	15.57	13.66	12.18	15.09	22.77	14.25	12.15	10.47	13.52
**Other**														
**BATXCRISP1**	31.18	27.60	22.88	27.19	30.25	29.20	33.18	22.08	22.05	25.45	21.79	25.24	25.70	27.04
**BATXHYAL1**	4.30	2.86	2.08	0.85	0.98	1.95	1.95	2.28	1.16	0.00	3.35	0.93	2.86	2.90
**BATXLAAO2**	10.75	31.41	29.12	27.19	51.72	26.28	20.49	36.54	64.99	60.28	47.77	56.08	48.54	13.52
**BATXNGF1**	0.00	3.81	5.20	5.10	2.93	5.84	6.83	3.81	5.80	9.38	5.03	0.93	6.66	6.76
**BATXNUC1**	3.23	8.57	30.16	19.55	24.40	9.73	13.66	12.94	18.57	34.83	16.76	25.24	23.79	8.69
**BATXPDE1**	39.78	41.88	45.75	34.84	40.99	28.23	35.13	27.40	34.82	30.81	33.52	29.91	46.64	45.38
**BATXVEGF5**	2.15	4.76	9.36	8.50	2.93	9.73	5.86	12.94	2.32	1.34	8.38	2.80	1.90	5.79

*Table includes only isoforms with more than 18 spectra in at least one of the venoms. Complete data is in Supplementary Table 1. Cells were formatted based on their values relative to the mean expression of all isoforms. Gradual scales in blue or red show values below or above average respectively.

### Correlation between venom proteome and patients’ signs and symptoms

We first attempted to correlate the levels of expression of venom protein families with the signs and symptoms presented by the corresponding patients and observed only a few positive correlations (Table 3; S3 Table). The abundance of SVMPs correlated to unclottable blood at admission (PII-class), edema and complications after 48 h (PI-class). SVSPs correlated only with complications after 48 h, and CTLs abundance correlated to edema (Table 3). SVMPs are recognized as key toxins in venoms of viper snakes, responsible for both local and systemic disturbances observed after envenomings [17], explaining the positive correlations observed. However, it is intriguing the lack of correlation between SVSPs and bleeding disturbances as SVSPs are thrombin-like enzymes involved in the consumption coagulopathy signed by unclottable blood at admission [33]. However, in this matter, the role of metalloproteases that act as factors II and X activators, identified in the venom of *B*. *atrox*, should be highlighted, which could explain the consumption coagulopathy [34,35]. Also, intriguing was the lack of correlation between hemorrhagic toxins, classified as PIII-class SVMPs, and local or systemic bleeding. Moreover, little is known about the relationship between CTLs and edema. Such unexpected results could be attributed to different isoforms within each protein family with distinct biological functions [24]. Thus, we proceeded with the comparisons of venom composition and correlations to patients’ signs and symptoms at the isoform level.

**Table 3:** Significant results of Spearman cross-correlation between ranks of normalized total spectrum counts venom protein families and patients’ symptoms*.

Isoforms	Severity of Envenomation	Unclottable Blood	Local Bleeding	Pain	Edema	Local Ecchymosis	Systemic Hemorrhage	Blister	Complications after 48 h
**CRISP**			-0.47 (p = 0.093)		-0.49 (p = 0.075)				
**CTL**					0.46 (p = 0.094)				
**HYAL**									
**LAAO**		-0.50 (p = 0.068)							
**NGF**									
**NUC**									
**PDE**									
**PLA2**	-0.53 (p = 0.051)				-0.61 (p = 0.020)				
**SVMP—I**					0.55 (p = 0.041)				0.49 (p = 0.074)
**SVMP—II**		0.57 (p = 0.032)							
**SVMP—III**			-0.47 (p = 0.093)						
**SVSP**									0.49 (p = 0.073)
**VEGF**									

Cells are formatted based on their correlation values with gradual scales in red or blue, corresponding to direct or inverse correlation, respectively.

As shown in Table 4, specific isoforms presented expression levels with statistically significant positive correlation to signs and symptoms presented by the patients. Local bleeding was correlated to the expression of the hemorrhagic toxin Batroxrhagin (BATXSVMPIII 28), also to a serine proteinase not yet functionally characterized, but that may display thrombin-like activity (BATXSVSP10) and to two CTLs (BATXCTL 9 and 28) that present 75–85% sequence identity with Bothrojaracin, an inhibitor of thrombin present in different venoms of *Bothrops* snakes [36]. These isoforms have already been correlated to bleeding processes in experimental models and our data validate these previous observations in signs and symptoms presented by human victims of snakebites. Interestingly, BATXPLA3, with 93% identity with a non-hemorrhagic myotoxin [37], also showed significant correlation with local bleeding. The positive correlation of BATXSVMPIII24 to edema and ecchymosis was also significant. This isoform presents 81% identity with Berythractivase, a non-hemorrhagic pro-coagulant SVMP from *B*. *erythromelas* venom that activates Factor II [38]. The only isoforms correlating to unclottable blood on admission were BATXSVMPIII9, a PIII-class SVMP with small identity to the already isolated toxins, and an isoform of vascular endothelial growth factor (BATXVEGF5). BATXSVMPIII9 also correlated to edema together with BATXSVMPIII27, also functionally uncharacterized. Some unexpected data were also observed as the highly significant correlation between BATXCTL23 with blister formation. This isoform presents 83% identity with a CTL isolated from *B*. *jararaca* venom that binds to the platelet receptor GPIb bp and inhibits platelet-aggregation [39]; however, the implications of CTLs with local effects of snake venoms as blistering are still uncertain. Similarly, some indications about the participation of CRISPs or LAAOs in blister formation are suggested here for the first time and deserve further attention. However, it is important to note that only one patient developed a blister indicating that these unexpected observations should be interpreted with caution. Envenomation severity and development of complications after 48 h correlated either positively or negatively with different toxins from the same families.

**Table 4:** Significant results of Cross-Correlation of Ranks (Spearman Correlation) between expression levels of predominant isoforms on venoms and patients’ symptoms*.

Isoforms	Severity of Envenomation	Unclottable Blood	Local Bleeding	Pain	Edema	Local Ecchymosis	Systemic hemorrhage	Blister	Complications after 48 h
**CTL**									
BATXCTL9			0.5 (p = 0.068)						
BATXCTL23								0.73 (p = 0.003)	
BATXCTL28	0.6 (p = 0.025)		0.64 (p = 0.013)						
BATXCTL39	0.57 (p = 0.034)								
**PLA**_**2**_									
BATXPLA2									
BATXPLA3	0.46 (p = 0.099)		0.46 (p = 0.099)						0.47 (p = 0.087)
BATXPLA5									
BATXPLA6						-0.46 (p = 0.099)			
**SVMP**									
BATXSVMPI5									
BATXSVMPIII1					-0.49 (p = 0.079)				
BATXSVMPIII2	-0.54 (p = 0.046)								-0.52 (p = 0.058)
BATXSVMPIII5									
BATXSVMPIII9	-0.48 (p = 0.083)	0.47 (p = 0.093)							
BATXSVMPIII16									
BATXSVMPIII18					0.47 (p = 0.092)				
BATXSVMPIII24					0.51 (p = 0.06)	0.67 (p = 0.009)			
BATXSVMPIII27	-0.53 (p = 0.05)		-0.54 (p = 0.046)		-0.46 (p = 0.099)				
BATXSVMPIII28	0.47 (p = 0.091)		0.5 (p = 0.068)						
**SVSP**									
BATXSVSP10	0.72 (p = 0.004)		0.61 (p = 0.021)						
BATXSVSP20									0.46 (p = 0.094)
**Other**									
BATXCRISP1								0.56 (p = 0.037)	
BATXHYAL1			-0.48 (p = 0.083)			-0.48 (p = 0.079)			
BATXLAAO2								0.62 (p = 0.017)	
BATXNGF1	0.57 (p = 0.034)								
BATXNUC1									
BATXPDE1									
BATXVEGF5		0.54 (p = 0.048)							0.47 (p = 0.087)

*Table includes only isoforms with more than 18 exclusive unique spectra counted in at least one of the venoms. Complete data is in Supplementary Table 3. Cells are formatted based on their correlation values with gradual scales in red or blue, corresponding to direct or inverse correlation, respectively.

Although we evidenced statistically significant correlation of individual toxins with patients’ signs and symptoms, these occurrences are certainly multifactorial, therefore, we attempted to proceed multivariate tests as multivariate linear least squares and multivariate logistic models. However, these tests failed to lead to any conclusion due to multicolinearity issues and mostly because of the small number of patients.

These are the first evidences correlating the venom proteome and signs and symptoms presented by snakebite patients. As previously predicted by experimental models [17], toxins included in the SVMP group presented the best correlation with many clinical manifestations. However, other isoforms included in the CTLs, SVSPs and other toxin groups also presented statistically significant correlations. These findings have a direct implication on the current discussion about the new generation snakebite treatments. Currently, antivenoms are composed of antibody molecules generated by immunization of large mammals with venom antigens. The basis for the neutralization of toxins by the polyclonal antibodies is their multiple specificities that make them able to bind and to neutralize most of the isoforms of the venom toxins. However, efforts have been made to substitute plasma collected from live animals in the antivenom manufacture’s process by monoclonal antibodies prepared in cell-culture media. However, monoclonal antibodies recognize a single epitope, usually restricted to specific isoforms. In this regard, to attempt a substitution of currently available antivenoms, pools with large types of monoclonal antibodies derived from different isoforms should be used. As shown here, different isoforms of toxins included in different toxin families correlated to signs and symptoms of snakebite. However, monoclonal antibodies are selective to specific motifs present in toxin molecules. Examples of monoclonal antibodies that recognize SVMPs from venoms of distinct species of *Bothrops* snakes are available but still, these antibodies recognize only homologous toxins in such venoms [40]. In this regard, to attempt a substitution of currently available polyclonal antivenoms, pools with monoclonal antibodies with specificity to different isoforms included in several protein families, should be used since, as shown here, distinct toxin isoforms correlated to symptoms of snakebite.

A more promising approach would be the use of enzyme inhibitors as an additional treatment of snakebites. In this regard, inhibitors of metalloproteinases are a very promising strategy as the catalytic site is well conserved within zinc-metalloproteinases, including SVMPs [41], which were shown here to present best correlations with clinical manifestations. Some metalloproteinase inhibitors, as batimastat, have already been tested [42,43] and trials involving new generation of SVMP inhibitors have been preconized by health authorities responsible for snakebite treatments. Phospholipase A_2_ inhibitors as Varespladib are also being recognized as additional first aid treatment in envenomings by some neurotoxic [44] or even coagulotoxic elapid venoms [45]. However, in the case of *Bothrops* venoms, particularly *B*. *atrox* snakes from Brazilian Amazon, phospholipases A_2_ are minor components and, as reported here, showed little correlation with signs and symptoms of envenomings. Nevertheless, other components as CTLs and SVSPs also play an important role in envenomings and the search of therapeutic inhibitors should also attempt neutralization of these usually neglected toxin groups.

Concluding, in this study we overcame the great difficulty to obtain the venom from the snakes inflicting 14 human envenomations. Although this is still a small number of samples, we were able to indicate that venom composition modulates signs and symptoms of snakebites, to confirm the prominent role of SVMPs and to include new possible toxin candidates to further attention in the treatment of patients.

## Supporting information

S1 Table Normalized number of spectra counted for each isoform during the shotgun analysis of the Bothrops atrox venom samples. (XLSX)

S2 Table Peptides identified during the shotgun analysis of the Bothrops atrox venom samples. (XLSX)

S3 Table Significant results of Cross-Correlation of Ranks (Spearman Correlation) between expression levels of venom isoforms and patients’ signs and symptoms. (PDF)

S1 Appendix STROBE Checklist. (DOC)
