# Supplementary material for: The relationship between clinics and the venom of the causative Amazon pit viper (Bothrops atrox)
Source: PLoS Negl Trop Dis. 2020 Jun 8;14(6):e0008299. doi: 10.1371/journal.pntd.0008299 (PMC7302866; doi:10.1371/journal.pntd.0008299)
Supplement: S3 Table — (PDF) [file pntd.0008299.s003.pdf]

Supplementary Table 3: Significant results of Cross-Correlation of Ranks (Spearman Correlation) between expression levels* of venom isoforms and patients symptoms.

Isoforms	Classification	Unclottable Blood	Local Bleeding	Pain	Edema	Ecchymosis	Systemic hemorrhage	Blister	Complications
BATXCRISP1									
BATXCTL1			0.58 (p=0.031)			0.56 (p=0.037)		0.47 (p=0.093)	0.53 (p=0.049)
BATXCTL2						0.51 (p=0.062)			
BATXCTL3	0.51 (p=0.064)								
BATXCTL4									
BATXCTL6									
BATXCTL9			0.5 (p=0.068)						
BATXCTL10			0.63 (p=0.017)	-0.52 (p=0.056)					
BATXCTL12									
BATXCTL13									
BATXCTL15			0.59 (p=0.025)						
BATXCTL18		-0.82 (p=0)				-0.54 (p=0.047)			
BATXCTL20								0.46 (p=0.1)	
BATXCTL22						-0.54 (p=0.047)			
BATXCTL23								0.73 (p=0.003)	
BATXCTL24									
BATXCTL25			-0.61 (p=0.02)						
BATXCTL26	-0.53 (p=0.051)		-0.54 (p=0.046)						
BATXCTL27									0.49 (p=0.073)
BATXCTL28	0.6 (p=0.025)		0.64 (p=0.013)						
BATXCTL29	-0.52 (p=0.058)	0.58 (p=0.031)	-0.47 (p=0.092)						
BATXCTL32	-0.48 (p=0.083)								
BATXCTL33									
BATXCTL34			0.46 (p=0.099)					0.48 (p=0.084)	
BATXCTL35			-0.68 (p=0.007)						
BATXCTL36								0.56 (p=0.037)	
BATXCTL37			-0.48 (p=0.083)			-0.48 (p=0.079)			
BATXCTL38								0.62 (p=0.017)	
BATXCTL39	0.57 (p=0.034)								
BATXCTL40									
BATXCTL42									
BATXCTL43			0.47 (p=0.093)						
BATXCTL44									
BATXCTL45									
BATXCTL50			-0.54 (p=0.047)						
BATXCTL53			0.56 (p=0.038)						
BATXCTL54									
BATXHYAL1									
BATXLAAO1									
BATXLAAO2		-0.68 (p=0.007)							
BATXNGF1									
BATXNUC1									
BATXPDE1									
BATXPLA1									
BATXPLA2									
BATXPLA3	0.46 (p=0.099)		0.46 (p=0.099)						0.47 (p=0.087)
BATXPLA5									
BATXPLA6						-0.46 (p=0.099)			
BATXSVMPI1					0.67 (p=0.009)				0.47 (p=0.091)
BATXSVMPI5									
BATXSVMPII1									
BATXSVMPII3						0.54 (p=0.046)			
BATXSVMPII4	-0.46 (p=0.097)	0.61 (p=0.021)			-0.47 (p=0.091)				
BATXSVMPII5	-0.53 (p=0.054)	0.47 (p=0.089)							
BATXSVMPII6	-0.6 (p=0.024)								
BATXSVMPII7									

BATXSVMPIII1					-0.49 (p=0.079)				
BATXSVMPIII2	-0.54 (p=0.046)								-0.52 (p=0.058)
BATXSVMPIII5									
BATXSVMPIII8									
BATXSVMPIII9	-0.48 (p=0.083)	0.47 (p=0.093)							
BATXSVMPIII10	-0.57 (p=0.033)		-0.46 (p=0.099)						
BATXSVMPIII11	-0.75 (p=0.002)				-0.56 (p=0.038)	-0.48 (p=0.079)			
BATXSVMPIII13	-0.64 (p=0.013)				-0.52 (p=0.056)	-0.55 (p=0.043)			
BATXSVMPIII14									
BATXSVMPIII15									
BATXSVMPIII16									
BATXSVMPIII17					-0.58 (p=0.031)				
BATXSVMPIII18					0.47 (p=0.092)				
BATXSVMPIII19						-0.48 (p=0.086)			
BATXSVMPIII21			0.68 (p=0.007)						
BATXSVMPIII22									
BATXSVMPIII24					0.51 (p=0.06)	0.67 (p=0.009)			
BATXSVMPIII25									
BATXSVMPIII26				-0.56 (p=0.037)					
BATXSVMPIII27	-0.53 (p=0.05)		-0.54 (p=0.046)		-0.46 (p=0.099)				
BATXSVMPIII28	0.47 (p=0.091)		0.5 (p=0.068)						
BATXSVSP1									
BATXSVSP2									
BATXSVSP3									-0.48 (p=0.079)
BATXSVSP6			0.47 (p=0.093)						
BATXSVSP7									
BATXSVSP9				-0.52 (p=0.056)					
BATXSVSP10	0.72 (p=0.004)		0.61 (p=0.021)						
BATXSVSP11		0.5 (p=0.071)							
BATXSVSP12									
BATXSVSP14	-0.64 (p=0.014)								
BATXSVSP16									
BATXSVSP17	0.52 (p=0.058)				0.67 (p=0.009)				
BATXSVSP19		0.54 (p=0.049)			0.56 (p=0.037)				0.53 (p=0.049)
BATXSVSP20									0.46 (p=0.094)
BATXVEGF2									
BATXVEGF5		0.54 (p=0.048)							0.47 (p=0.087)

* Expression levels of individual isoforms were calculated as Exclusive Unique Spectrun Counts using the Scaffold package.
